# The IgH 3′ regulatory region governs μ chain transcription in mature B lymphocytes and the B cell fate

**DOI:** 10.18632/oncotarget.3010

**Published:** 2015-03-03

**Authors:** Alexis Saintamand, Pauline Rouaud, Armand Garot, Faten Saad, Claire Carrion, Christelle Oblet, Michel Cogné, Eric Pinaud, Yves Denizot

**Affiliations:** ^1^ CNRS, CRIBL, UMR 7276, Limoges, France; ^2^ Université de Limoges, CRIBL, UMR 7276, Limoges, France; ^3^ Institut Universitaire de France, Paris, France

**Keywords:** BCR, B cell fate, IgH 3′ regulatory enhancers, knock-out mice

## Abstract

We report that the IgH 3′ regulatory region (3′RR) has no role on μ chain transcription and pre-BCR expression in B cell progenitors. In contrast, analysis of heterozygous IgH a^Δ3′RR^/b^wt^ mice indicated that the 3′RR controls μ chain transcripts in mature splenocytes and impacts membrane IgM density without obvious effect on BCR signals (colocalisation with lipid rafts and phosphorylation of Erk and Akt after BCR crosslinking). Deletion of the 3′RR modulates the B cell fate to less marginal zone B cells. In conclusion, the 3′RR is dispensable for pre-BCR expression and necessary for optimal commitments toward the marginal zone B cell fate. These results reinforce the concept of a dual regulation of the IgH locus transcription and accessibility by 5′ elements at immature B cell stages, and by the 3′RR as early as the resting mature B cell stage and then along further activation and differentiation.

## INTRODUCTION

Lymphopoiesis is coupled with programmed accessibility of Ig genes to transcription and to several major transcription-dependent DNA remodelling events [1, 2]. While 5′ *cis*-regulatory elements (Eμ and IGCR1) control V(D)J recombination [2–4], the IgH 3′ regulatory region (3′RR) controls class switch recombination (CSR) [2, 5] and IgH somatic hypermutation (SHM) [6]. B cells are continuously instructed by B cell receptor (BCR) signals to make crucial cell fate decisions at several checkpoints during their development [7, 8]. Such an important choice made by immature B cells is to become either a follicular (FO) or a marginal zone (MZ) B cell. Influence of the BCR strength on B cell fate has been investigated by various experimental approaches (such as mutations affecting BCR components and proteins implicated in BCR signalling) both affecting pre-BCR and BCR signals and thus resulting in multiple anomalies at various stages of B cell maturation [7, 8]. The commonly accepted hypothesis is that the BCR strength controls B cell fate with weak BCR signalling inducing MZ B cell development while strong BCR signalling favours the development of FO B cells [7, 8]. We studied heterozygous IgH a^Δ3′RR^/b*^wt^* mice. We report that the 3′RR has no role on the pre-BCR expression but governs μ gene transcription and thus BCR expression specifically in mature B cells where its deletion affects the B cell fate toward less MZ B cells.

## RESULTS AND DISCUSSION

### Expression of a 3′RR-deleted allele in bone marrow B cells

**Figure 1:**
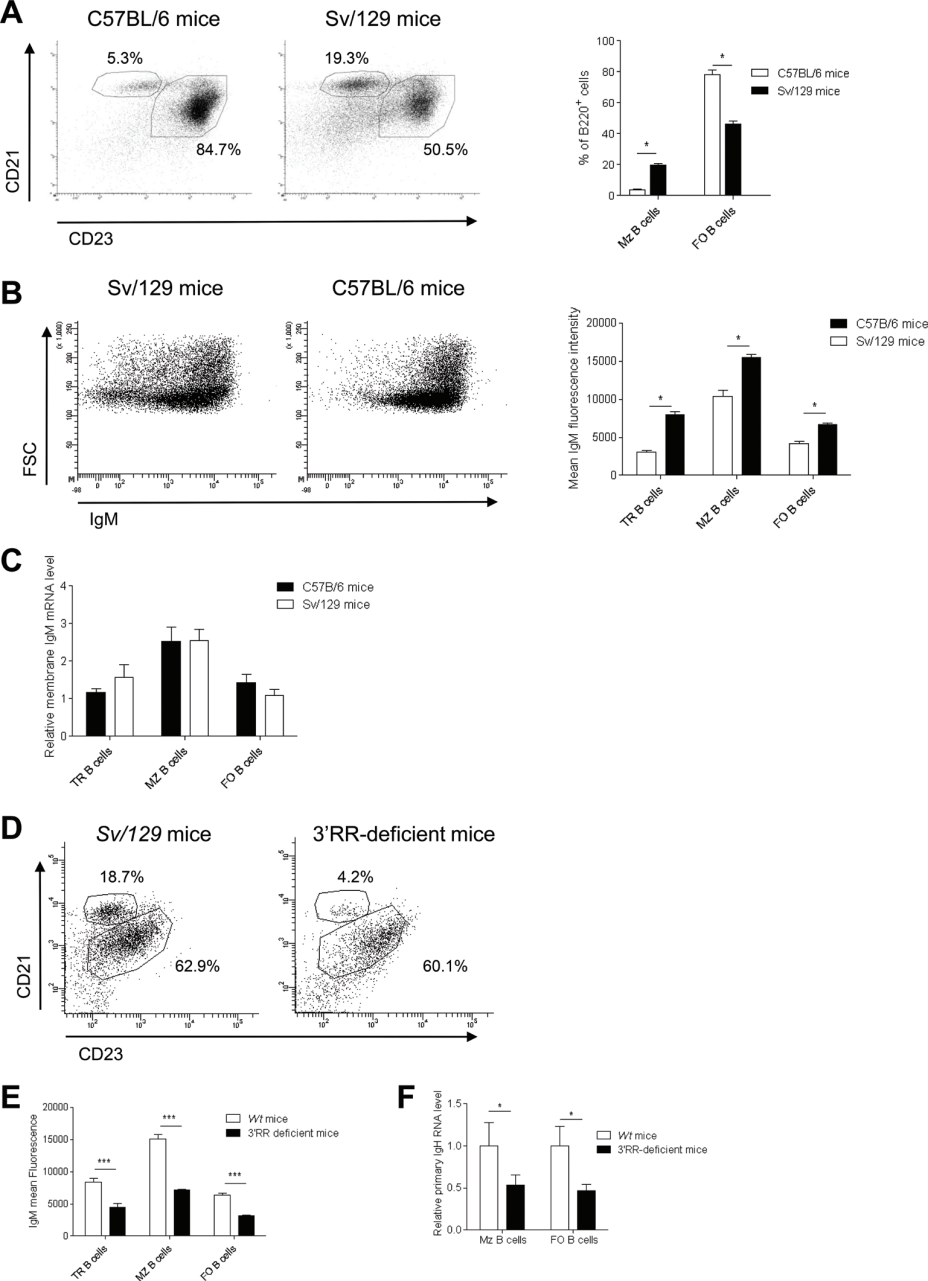
B cell fate and IgM expression in C57BL/6 and Sv/129 mice **(A)** Left part - flow cytometry analysis of follicular (FO) B cells (B220^+^CD21^low^CD23^high^) and marginal zone (MZ) B cells (B220^+^CD21^high^CD23^low^) in spleen of C57BL/6 (IgH b*^wt^*/b*^wt^*) and Sv/129 (IgH a*^wt^*/a*^wt^*) *wt* mice. Cells were gated on B220^+^ cells. One representative experiment of three Sv/129 and seven C57BL/6 mice is shown. Right part - percentage of splenic FO and MZ B cells. Mean ± SEM of three and seven values for Sv/129 and C57BL/6 mice, respectively. *p < 0.05 (Mann-Whitney *U*-test). **(B)** Left part - flow cytometry analysis of B220^+^IgM^+^ B cells in spleen of C57BL/6 and Sv/129 mice. One representative experiment of three Sv/129 and seven C57BL/6 mice is shown. Cells were gated on B220^+^ cells. The anti-IgM labelled antibody binds both the *a* and the *b* allotypes. Right part - Mean IgM intensities on FO, MZ and transitional (TR, B220^+^AA4.1^+^) B cells in spleen of Sv/129 and C57BL/6 mice. Mean ± SEM of three and seven values for Sv/129 and C57BL/6 mice, respectively. *p < 0.05 (Mann-Whitney *U*-test). **(C)** Similar μ transcription in sorted TR, FO and MZ B cells of Sv/129 and C57BL/6 mice. Mean ± SEM of 5 mice. Values were normalized to GAPDH transcripts. **(D)** Flow cytometry analysis of FO and MZ B cells in spleen of homozygous 3′RR-deficient mice (IgH a^Δ3′RR^/a^Δ3′RR^) and Sv/129 (IgH a*^wt^*/a*^wt^*) mice. Cells were gated on B220^+^ cells. One representative experiment out of six is shown. **(E)** Percentages of splenic TR, FO and MZ B cells in 3′RR-deficient mice and Sv/129 mice. Mean ± SEM of six mice. *p < 0.001 (Mann-Whitney *U*-test). **(F)** Real time PCR analysis of primary IgH transcripts in sorted FO and MZ B cells of 3′RR-deficient mice and Sv/129 mice. Mean ± SEM of five mice. *p < 0.05 (Mann-Whitney *U*-test). Values were normalised to GAPDH transcripts.

**Figure 2:**
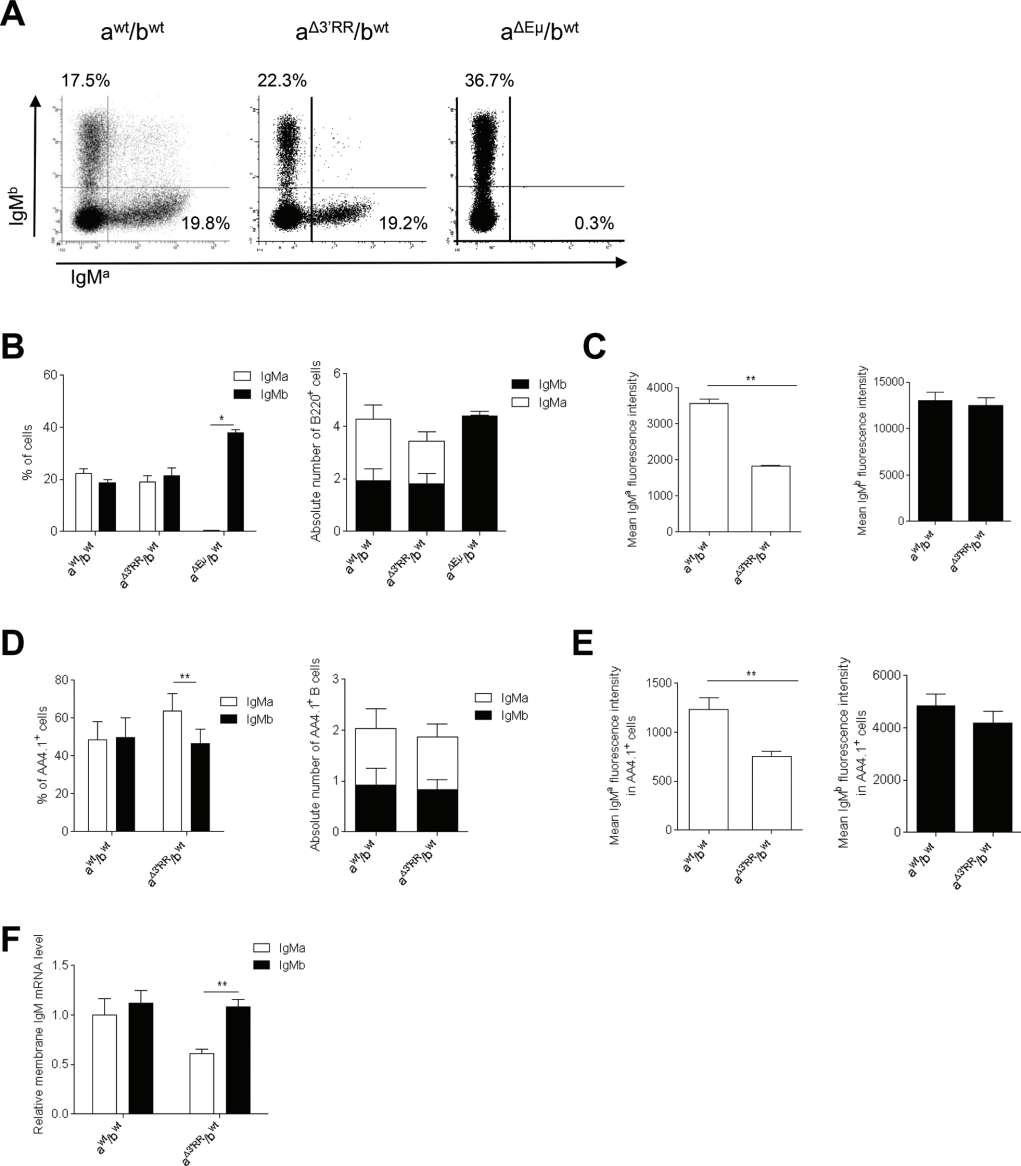
Expression of a 3′RR-deleted allele in bone marrow B cells **(A)** Flow cytometry analysis of IgM^a^ and IgM^b^ on B220^+^ bone marrow cells of heterozygous a^Δ3′RR^/b*^wt^*, a*^wt^*/b*^wt^* and a^ΔEμMARs^/b*^wt^* mice. One representative experiment out of ten is shown for a^Δ3′RR^/b*^wt^* and a*^wt^*/b*^wt^* mice. One representative experiment out of four is shown for a^ΔEμMARs^/b*^wt^* mice. Cells were gated on B220^+^ cells. **(B)** Percentages (left part) and numbers (right part) of B220^+^ bone marrow B cells expressing the *a* or *b* allele in a^Δ3′RR^/b*^wt^*, a*^wt^*/b*^wt^* and a^ΔEμMARs^/b*^wt^* mice. Mean ± SEM of ten a^Δ3′RR^/b*^wt^* mice, ten a*^wt^*/b*^wt^* and four a^ΔEμMARs^/b*^wt^* mice. Millions of bone marrow B cells are reported. *p < 0.05 (Mann-Whitney *U*-test). **(C)** IgM^a^ and IgM^b^ fluorescence intensity on B220^+^ B cells of a^Δ3′RR^/b*^wt^* and a*^wt^*/b*^wt^* mice. Mean ± SEM of ten mice. **p < 0.01 (Mann-Whitney *U*-test). **(D)** Percentages (left part) and numbers (right part) of B220^+^AA4.1^+^ bone marrow B cells in a^Δ3′RR^/b*^wt^* and a*^wt^*/b*^wt^* mice. Mean ± SEM of ten mice. **p < 0.01 (Mann-Whitney *U*-test). **(E)** IgM^a^ and IgM^b^ fluorescence intensity on B220^+^AA4.1^+^ bone marrow B cells in a^Δ3′RR^/b*^wt^* and a*^wt^*/b*^wt^* mice. Mean ± SEM of ten mice. **p < 0.01 (Mann-Whitney *U*-test). **(F)** Real time PCR analysis of μ membrane transcripts in sorted B220^+^AA4.1^+^ bone marrow B cells of a^Δ3′RR^/b*^wt^* and a*^wt^*/b*^wt^* mice. Values were normalized to GAPDH transcripts. Mean ± SEM of six mice. *p < 0.05 (Mann-Whitney *U*-test).

Mouse substrains have dissimilar differentiation programs culminating in different B cell fate and BCR expression [9] (Figure 1). To assess B cell differentiation issues linked to genetic background, our study was carried out in heterozygous IgH a^Δ3′RR^/b*^wt^* mice, compared to *wt* F1 IgH a*^wt^*/b*^wt^* mice. Analysis of bone marrow B cells with IgM-allotype specific antibodies indicated similar percentages and numbers of B cells expressing either *a* or *b* allotype in a^Δ3′RR^/b*^wt^* and a*^wt^*/b*^wt^* mice; as a negative control IgM^a^-expressing B cells were not detected in mice carrying heterozygous deletion of the Eμ region (a^ΔEμ^/b*^wt^*) [10] (Figure 2A and 2B). The mean membrane IgM^a^ (but not IgM^b^) density was reduced in heterozygous a^Δ3′RR^/b*^wt^* compared to a*^wt^*/b*^wt^* mice (Figure 2C). Analysis of immature B220^+^AA4.1^+^ B cells indicated a slight increase of the percentage (but not the numbers) of cells expressing the *a* allotype in heterozygous a^Δ3′RR^/b*^wt^* mice (Figure 2D). A decreased membrane IgM^a^ (but not IgM^b^) density was found in heterozygous a^Δ3′RR^/b*^wt^* mice compared to a*^wt^*/b*^wt^* mice (Figure 2E). Finally, real time PCR analysis indicated a reduced transcription of the *a* allele (but not *b*) in B220^+^AA4.1^+^ sorted B cells from a^Δ3′RR^/b*^wt^* mice compared to a*^wt^*/b*^wt^* mice (Figure 2F). Taken altogether these results are indications that the 3′RR-deficient allele underwent V(D)J recombination at a rate and a timeframe similar to the *wt* allele. Indeed any delay in IgH chain expression from the mutated allele would be expected to result in unbalanced expression of IgH alleles in immature B cells from heterozygous mice (as found with the a^ΔEμ^ allele) [10]. Bone marrow IgMa^Δ3′RR^ B cells had a lower IgH transcription and membrane IgM expression confirming an early 3′RR transcriptional control immediately after the pre-B cell stage [11]. The slight accumulation of newly formed IgMa^Δ3′RR^ B cells may imply a B cell fate decision defect.

### Expression of a 3′RR-deleted allele in peripheral B cells

**Figure 3:**
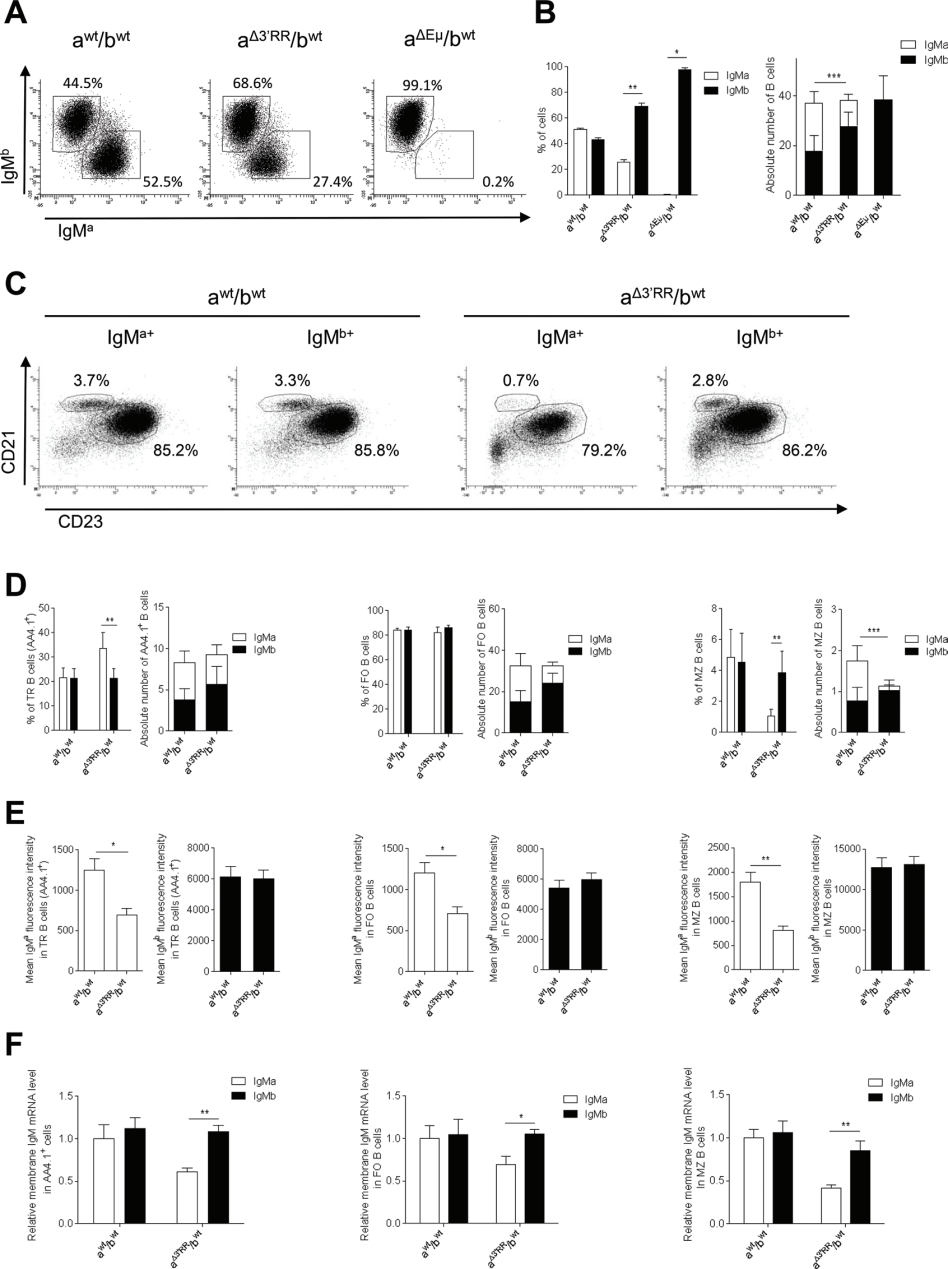
Expression of a 3′RR-deleted allele in splenic B cells **(A)** Flow cytometry analysis of IgM^a^ and IgM^b^ in spleen of heterozygous a^Δ3′RR^/b*^wt^*, a*^wt^*/b*^wt^* and a^ΔEμMARs^/b*^wt^* mice. One representative experiment out of ten is shown for a^Δ3′RR^/b*^wt^* and a*^wt^*/b*^wt^* mice. One representative experiment out of four is shown for a^ΔEμMARs^/b*^wt^* mice. Cells were gated on B220^+^ cells. **(B)** Percentages (left part) and numbers (right part) of B220^+^ splenic B cells expressing the *a* or *b* allele in a^Δ3′RR^/b*^wt^*, a*^wt^*/b*^wt^* and a^ΔEμMARs^/b*^wt^* mice. Mean ± SEM of ten a^Δ3′RR^/b*^wt^* mice, ten a*^wt^*/b*^wt^* and four a^ΔEμMARs^/b*^wt^* mice. Millions of splenic B cells are reported. The significance is for IgM^a^ cells in the right part. *p < 0.05, **p < 0.01, **p < 0.01 (Mann-Whitney *U*-test). **(C)** Flow cytometry analysis of follicular (FO) B cells (B220^+^CD21^low^CD23^high^) and marginal zone (MZ) B cells (B220^+^CD21^high^CD23^low^) in a^Δ3′RR^/b*^wt^* and a*^wt^*/b*^wt^* mice. One representative experiment out of ten is shown. Cells were gated on B220^+^ cells. **(D)** Percentages and numbers (in millions) of TR (AA4.1^+^), FO and MZ B cells in a^Δ3′RR^/b*^wt^* and a*^wt^*/b*^wt^* mice. Mean ± SEM of ten mice. Million numbers of cells are reported. The significance is for MZ IgM^a^ cells in the right part. **p < 0.01 (Mann-Whitney *U*-test). **(E)** IgM^a^ and IgM^b^ densities on TR, FO and MZ B cells in a^Δ3′RR^/b*^wt^* and a*^wt^*/b*^wt^* mice. Mean ± SEM of ten mice. *p < 0.05, **p < 0.01 (Mann-Whitney *U*-test). **(F)** μ transcription in sorted TR, FO and MZ B cells of a^Δ3′RR^/b*^wt^* and a*^wt^*/b*^wt^* mice. Mean ± SEM of 6 mice. Values were normalized to GAPDH transcripts. *p < 0.05, **p < 0.01 (Mann-Whitney *U*-test).

**Figure 4:**
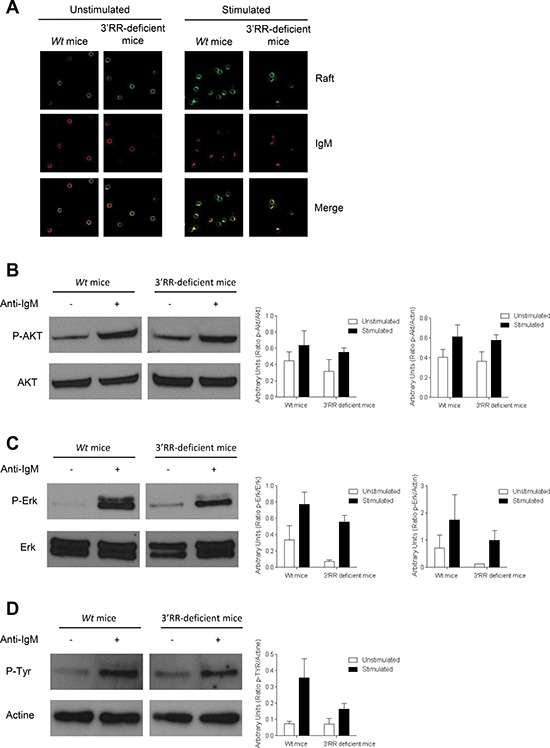
BCR signalling response in 3′RR-deficient B splenocytes **(A)** BCR and lipid rafts colocalization experiments. 3′RR deficient mice (a^Δ3′RR^/a^Δ3′RR^) and Sv/129 *wt* mice (a*^wt^*/a*^wt^*) were investigated. CD43^−^ B cells were stained with cholera toxin Alexa 488 followed by stimulation with anti-IgM Alexa 594 labelled antibodies. Arrows point to areas where lipid rafts colocalise with membrane BCR. One representative experiment out of four is shown. **(B)** BCR signalling in 3′RR-deficient mice. CD43^−^ splenic B cells (3 × 106 cells/ml) from *wt* Sv/129 mice (a*^wt^*/a*^wt^*) and 3′RR-deficient mice (a^Δ3′RR^/a^Δ3′RR^) (were stimulated with 15 μg/ml of anti-IgM at 37°C for 10 min. Western Blot experiments were performed with 30 μg of proteins. Actin was used as an internal loading control. phosphor-Akt and Akt were investigated with specific anti-phosphor-Akt and anti-Akt antibodies, respectively. One representative experiments out of 3. Relative intensity of phosphor-Akt bands were related to Akt and actin bands (located in D). **(C)** phosphor-Erk and Erk were investigated with specific anti-phosphor-Erk and anti-Erk antibodies, respectively. One representative experiments out of 3. Relative intensity of phosphor-Erk bands were related to Erk and actin bands (located in D). **(D)** Total phosphor-Tyr was investigated with specific anti-phosphor-Tyr antibodies. One representative experiments out of 3. Relative intensity of phosphor-Tyr bands were related to actin bands. No significant differences for p-Akt, Akt, p-Erk, Erk and p-Tyr between 3′RR-deficient mice and *wt* mice (Mann-Whitney *U*-test).

In contrast to bone marrow B-lineage cells, a strong disadvantage of the mutated *a* allotype finally manifested in mature splenic B cells of IgH a^Δ3′RR^/b*^wt^* mice (Figure 3A–3C). This precisely identified the transition from immature to mature B cells as the time point where the 3′RR-deficiency altered B cell differentiation and introduced a biased representation of the mutant IgH allele. In a^Δ3′RR^/b*^wt^* mice, proportion (but not numbers) of splenic IgM^a^AA4.1^+^ transitional (TR) B cells was increased when compared to a*^wt^*/b*^wt^* mice (Figure 3D, left part). Deletion of the 3′RR had no effect on FO B cells (B220^+^CD21^low^CD23^high^) (Figure 3D, middle part) while a marked reduction of MZ B cells (B220^+^CD21^high^CD23^low^) was found (Figure 3E, right part). The mean IgM^a^ (but not IgM^b^) intensity was significantly reduced in a^Δ3′RR^/b*^wt^* compared to a*^wt^*/b*^wt^* mice in TR, FO and MZ B cells (Figure 3E). Real time PCR analysis showed a reduced IgM^a^ (but not IgM^b^) transcription in sorted TR, FO and MZ B cells from a^Δ3′RR^/b*^wt^* (Figure 3F). Thus, deletion of the 3′RR affected membrane IgM expression in mature B cell and the B cell fate toward the MZ phenotype. These results markedly contrast with the lower FO phenotype resulting from hypomorphic expression of either Igα or of an IgH-chain allele, both affecting pre-BCR and BCR expression and resulting in multiple anomalies at various stages of B cell maturation [12, 13]. The cell fate decision made by immature B cells to become a FO or a MZ B cell is controlled by the BCR strength [7, 8]. We investigated BCR signalling in homozygous a^Δ3′RR^/a^Δ3′RR^ mice that had lower levels of MZ B cells and IgM density than in a*^wt^*/a*^wt^* Sv/129 mice (Figure 1). Engagement of BCR with anti-IgM stimulation efficiently induced the co-localisation between BCR and lipid rafts in 3′RR-deficient splenocytes (Figure 4) that is a prerequisite for an efficient BCR signalling [14, 15]. We show that surface Ig cross-linking induces amounts of BCR signalling (Erk and Akt phosphorylations) in 3′RR-deficient B cells without obvious differences to *wt* mice (Figure 4).

## CONCLUSION

Deletion of the 3′RR had no impact on bone marrow B cell populations on the use of a 3′RR-deficient allele in competition with a *w*t allele. This reinforced studies highlighting the key role of 5′ IgH locus regulatory elements in recombination and transcription at immature stages leading to pre-BCR expression [2, 3, 10, 16]. In contrast, a late developmental defect appears in peripheral B lymphocytes with an impact of the 3′RR deletion on IgH chain expression. Whether several models reported that lowered tonic BCR signalling alters B cell fate by favouring the development of MZ B cells in the detriment of FO B cells [7, 8], other studies do not [17, 18]. We show that deletion of the 3′RR lowered IgH transcription, BCR density, and BCR signalling with consequences on B cell fate, disadvantaging MZ subset in favour to FO B cells. This suggests that B cell fate might not be only governed by the BCR tonic signal but relies on fine IgH chain tuning, by multiple regulatory elements including the 3′RR, at specific stages of B cell development. While 5′ IgH elements solely control IgH (and thus pre-BCR) expression at immature stages, the 3′RR comes into IgH (and thus BCR) transcriptional control in mature B cells.

## MATERIALS AND METHODS

### Mice

Our research has been approved by our local ethics committee review board (Comité Régional d'Ethique sur l'Expérimentation Animale du Limousin, Limoges, France) and carried according the European guidelines for animal experimentation. The disruption of the 3′RR was carried out in a Sv/129 embryonic stem cell line [5]. Mice were bred and maintained under specific pathogen-free conditions. Age-matched littermates (8 weeks old) were used in all experiments.

### Cell cytometry analysis

Cells were incubated with anti-B220-BV510, anti-CD21-BV650, anti-CD23-PC7, anti-IgM-APC, anti-IgMa-FITC, anti-IgMb-PE, anti-CD19-APCH7 and anti-AA4.1-APC antibodies (Southern Biotechnologies and Beckton Dickinson) and analysed on a Fortessa LSR2 (Beckton Dickinson) [19–21]. Heterozygous IgH a^Δ3′RR^/b*^wt^* mice generated by crossing homozygous 3′RR-deficient mice (IgH a^Δ3′RR^/a^Δ3′RR^) with C57BL/6 mice (IgH b*^wt^*/b*^wt^*) were investigated. Mixed Sv/129 × C57BL/6 mice (IgH a*^wt^*/b*^wt^*) were used as control mice.

### Transcript analysis

Bone marrow B220^+^AA4.1^+^IgMa^+^ and B220^+^ AA4.1^+^IgMb^+^ cells were sorted with specific anti-B220, anti-IgMa, anti-IgMb and anti-AA4.1 labelled antibodies using a BD FACSAria III. Splenic follicular (FO) B cells (B220^+^CD21^low^CD23^high^), marginal zone (MZ) B cells (B220^+^CD21^high^CD23^low^) and transitional (TR) B cells (B220^+^AA4.1^+^) were sorted with specific anti-B220-, anti-CD21-, anti-CD23 and anti-AA4.1 labelled antibodies. Total RNA was extracted and real time PCR was performed in duplicate by using TaqMan and SYBR assay reagents and analysed on an ABI Prism 7000 system (Applied Biosystems) [22]. μ membrane forward (in exon μ 4): 5′-TGGAACTCCGGAGAGACCTA-3′; μ membrane reverse (in exon μ membrane 1): 5′-TTCCTCCTCAGCATTCACCT-3′. GAPDH was used for normalization of gene expression levels (reference Mm99999915-g1). Heterozygous IgH a^Δ3′RR^/b*^wt^* mice and mixed Sv/129 × C57BL/6 mice (IgH a*^wt^*/b*^wt^*) were used for cell sorting experiments.

### Western blot analysis

CD43^−^ B splenocytes (15 × 10^6^) were stimulated with 15 μg of goat anti-mouse κ (Southern Biotech) for 10 min at 37°C. Cell lysates (30 μg) were analysed by SDS-PAGE and transferred onto polyvinylidene difluoride membranes (Millipore) as previously reported [22, 23]. After blocking, membranes were incubated with anti-phosphor-Tyr (Cell signalling), anti-phosphor-Erk (Cell signalling), anti-Erk (Cell signalling), anti-phosphor-Akt (Cell signalling), anti-Akt (Cell signalling) and anti-actin antibodies (Sigma), and revealed with HRP-labelled goat anti-rabbit Ig (Biorad) by chemoluminescence (ECL plus, Amersham). Results were analysed with the Java-based image processing program ImageJ that can calculate area and pixel value statistics of user-defined selections and intensity thresholded objects. Homozygous IgH a^Δ3′RR^/a^Δ3′RR^ and Sv/129 mice (IgH a*^wt^*/a*^wt^*) were used for western blot experiments.

### Lipid raft aggregation

For BCR and lipid raft colocalization experiments, splenic B cells were purified by hypotonic lysis of red blood cells followed by incubation with anti-CD43-coated MicroBeads (Miltenyi Biotec), which bind to all splenic cells except resting mature B cells. B cells were obtained by passing the cells through a negative depletion column attached to an OctoMACS magnet (Miltenyi Biotec). 1 × 10^6^ CD43^−^ B splenocytes (100 μl) were stained with 80 ng of cholera toxin B, a marker of lipid rafts, conjugated to Alexa 488 and 20 μg of goat anti-mouse IgM conjugated to Alexa 594 (Molecular Probes) for 30 min on ice then 5 min at 37°C for the stimulated conditions. Cells were then fixed with 4% PFA. For non stimulated conditions cells were incubated 30 min with an anti-IgM-PE on ice and analysed with a Zeiss LSM 510 META confocal microscope. Homozygous IgH a^Δ3′RR^/a^Δ3′RR^ and Sv/129 mice (IgH a*^wt^*/a*^wt^*) were used for experiments.
